# Chemical screening identifies ROCK1 as a regulator of migrasome formation

**DOI:** 10.1038/s41421-020-0179-6

**Published:** 2020-08-04

**Authors:** Puzhong Lu, Rui Liu, Di Lu, Yue Xu, Xueyi Yang, Zheng Jiang, Chun Yang, Li Yu, Xiaoguang Lei, Yang Chen

**Affiliations:** 1 The State Key Laboratory of Membrane Biology, Tsinghua University-Peking University Joint Center for Life Sciences, School of Life Sciences, Tsinghua University, 100084 Beijing, China; 2 Beijing National Laboratory for Molecular Sciences, Key Laboratory of Bioorganic Chemistry and Molecular Engineering of Ministry of Education, College of Chemistry and Molecular Engineering, Peking-Tsinghua Center for Life Science, Peking University, 100871 Beijing, China; 3 Institute of Biomechanics and Medical Engineering, School of Aerospace Engineering, Tsinghua University, 10084 Beijing, China; 4 Center for Precision Medicine Multi-Omics Research, Peking University Health Science Center, Peking University, 100191 Beijing, China

**Keywords:** Organelles, Cell migration

Dear Editor,

Migrasomes are newly discovered cellular organelles, first described in 2015^1,2^. Migrasomes are vesicles with diameters of 0.5–3 µm which are generated during cell migration. Cellular contents such as cytosolic components are actively transported to migrasomes and eventually released extracellularly. Thus, migrasomes are proposed as a mechanism for cell–cell communications. Migrasomes are essential for organ morphogenesis during zebrafish embryonic development^3^. Moreover, it has been shown that migrasomes are detected in human serum^4^. Assembly of tetraspanin- and cholesterol-enriched membrane microdomains into micron-scale macrodomains are necessary and sufficient for migrasome formation^5^. In addition, integrins provide the adhesion force for retraction fiber tethering, which are pivotal in migrasome biogenesis process^6^. Pairing of integrins with specific ECM partners for proper adhesion is a determinant for migrasome formation. So far, the systematic studies on detailed regulatory mechanisms of migrasome biogenesis are still lacking.

We designed a chemical genetic screening to identify chemical compounds and their protein targets which interfered with migrasome formation. We used NRK cells stably expressing TSPAN4-GFP to generate migrasomes in 96-well plates and treated with compounds. A diagram of the workflow used for screening is shown in Fig. 1a. Image acquisition was achieved automatically. To assay migrasome generation, the number of cells and migrasomes was quantified and the average migrasome number per cell was calculated. It has been reported that fibronectin (FN) promotes migrasome formation^2^. Using our assay, we tested the effect of increasing the concentration of fibronectin. The average migrasome number per cell increased as the fibronectin concentration increased (Supplementary Fig. S1a). GLPG0187 is the inhibitor of integrin α5β1, which is essential for migrasome biogenesis. GLPG0187 inhibited migrasome biogenesis in a concentration-dependent manner without cytotoxicity (Supplementary Fig. S1b). Based on these results, we concluded that the assay was sufficiently robust and we proceeded with high-throughput screening. We performed the assay with 2240 compounds at a concentration of 10 µM in a 96-well plate format. We identified 507 compounds which had significant inhibitory effect on migrasome generation (Fig. 1b). Indeed, we found that 463 out of the 507 hits showed no or less retraction fibers indicating defect of cell migration (Fig. 1b, Supplementary Fig. S1c). This is a confirmation of the notion that migrasome formation is migration dependent^2^. We focused on the 12 candidates which show significant decreased migrasome number with relatively normal retraction fiber (Fig. 1b, Supplementary Fig. S1c). We performed secondary screening of the 12 candidates. SAR407899 showed stable inhibition of migrasome formation without cytotoxicity or impaired cell proliferation (Fig. 1c, d). The number of migrasomes/100 μm was also significantly reduced compared to DMSO-treated cells (Fig. 1e), which excluded the effect of retraction fiber and cell migration on migrasome formation.

**Fig. 1:**
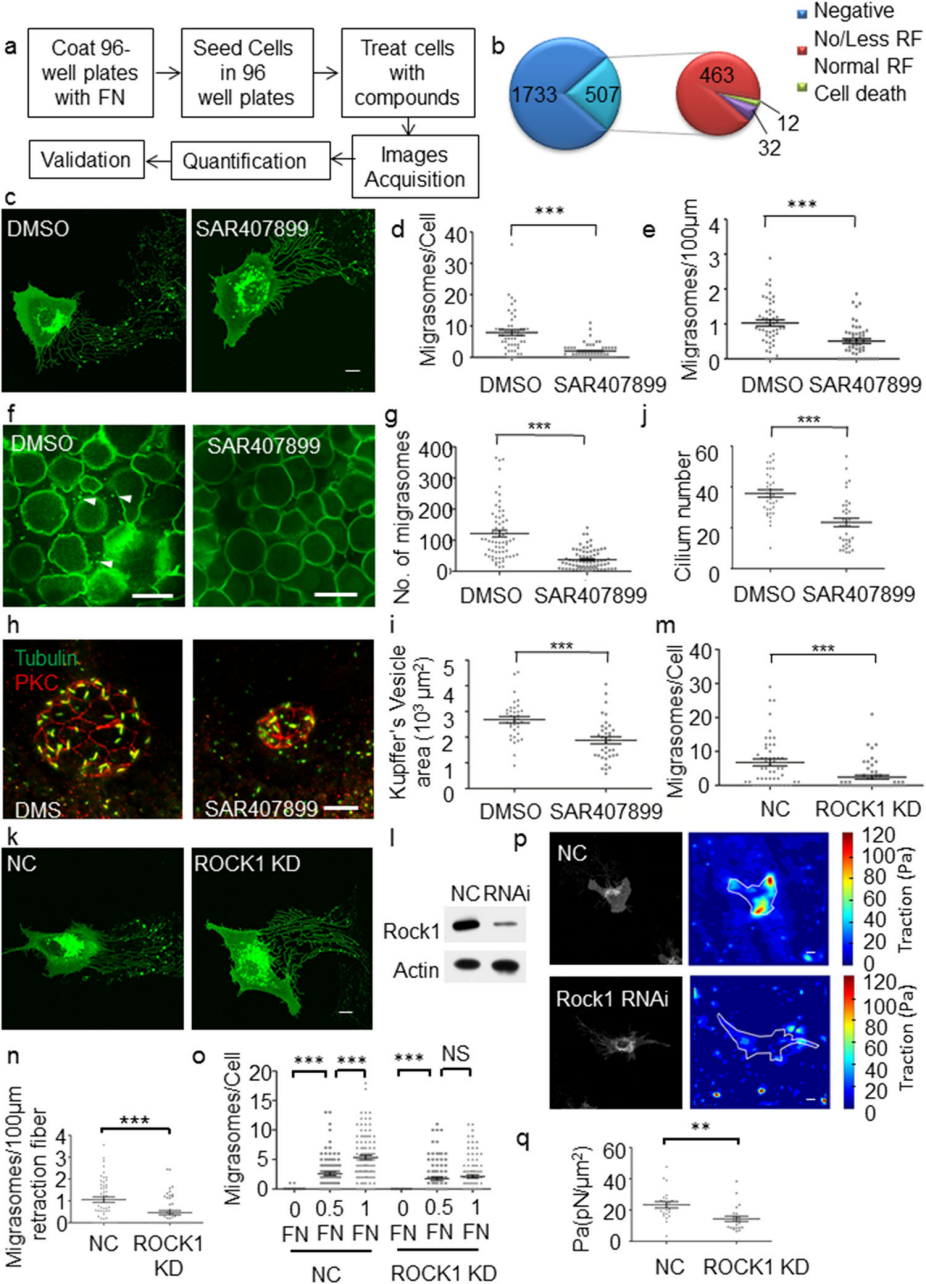
Chemical screening identifies ROCK1 as a regulator of migrasome formation. **a** Schematic illustration of the procedure for the high-throughput phenotypic screening. **b** Pie chart showing the number of compounds in each phenotype categories illustrated in Supplementary Fig. S1c. **c** Representative confocal images of TSPAN4-GFP-expressing NRK cells treated with DMSO or 10 μM SAR407899. Scale bar, 10 μm. **d** Quantification of the average migrasome number per cell from **c** (mean ± s.e.m.). *n* = 50 cells. ***P < 0.001. **e** Quantification of the average migrasome number per 100 μm retraction fiber from **c** (mean ± s.e.m.). *n* = 50 cells. ***P < 0.001. **f** Representative images of zebrafish embryos treated with DMSO or SAR407899. Migrasomes were labeled with PH–GFP and visualized by spinning disk. Arrowheads indicate migrasomes. Scale bar, 50 μm. **g** Quantification of the migrasomes number in zebrafish embryos from **f** (mean ± s.e.m.). Embryos from three independent experiments were pooled for quantification. DMSO, *n* = 67; SAR407899, *n* = 76. ***P < 0.001. **h** Embryos treated with either DMSO or SAR407899 were analyzed at the six-somite stage for KV formation by immunostaining with anti-PKC and anti-Tubulin antibodies. *Z*-stack images were acquired by confocal microscopy. Scale bar, 20 μm. **i** Quantification of KV size from **h**. Embryos from three independent experiments were pooled for quantification (mean ± s.e.m.). DMSO, *n* = 40; SAR407899, *n* = 40. ***P < 0.001. **j** Quantification of the number of cilia from **h**. Embryos from three independent experiments were pooled for quantification (mean ± s.e.m.). DMSO, *n* = 40; SAR407899, *n* = 40. ***P < 0.001. **k** NRK cells expressing TSPAN4-GFP were transfected with control (NC) or ROCK1 RNAi. Representative images of cells with each treatment are shown. Scale bar, 10 μm. **l** The RNAi knockdown efficiency was determined by western blot using antibodies against ROCK1. **m** Quantification of the average migrasome number per cell of cells from **k** (mean ± s.e.m.). NC, *n* = 50; ROCK1 KD, *n* = 50 cells. ***P < 0.001. **n** Quantification of average number of migrasomes/100 μm retraction fiber of cells from **k** (mean ± s.e.m.). NC, *n* = 50 cells; ROCK1 KD, *n* = 50 cells. ***P < 0.001. **o** WT or ROCK1 knockdown NRK cells were plated on culture plates coated with different concentration of fibronectin (FN). 0 FN indicates no FN coated. 0.5 FN indicates the plates were coated with 5 μg/ml FN. 1 FN indicates the plates were coated with 10 μg/ml FN. The average migrasome number per cell was quantified (mean ± s.e.m.). *n* = 100 cells for each treatment. ***P < 0.001. **p** Representative confocal images of TSPAN4-GFP-expressing NRK cells treated with control (NC) or ROCK1 siRNAs and the corresponding maps of the reconstructed traction forces were shown. Scale bar, 10 μm. **q** Quantification of the average traction of TSPAN4-GFP-expressing NRK cells treated with NC or ROCK1 siRNAs from **p** (mean ± s.e.m.). NC, *n* = 30 cells; ROCK1 KD, 30 cells. **P < 0.01.

In zebrafish embryos, generation of migrasomes has been observed during gastrulation. Migrasomes were shown to be essential for organ morphogenesis during embryonic development^3^. We thus tested the inhibitory effect of SAR407899 on migrasome biogenesis in a zebrafish model. For this purpose, we overexpressed PH domain protein tagged with GFP to label migrasomes in embryos. We took images of the entire embryo with 25 *Z*-stack images at 2 μm intervals (Supplementary Fig. S2). The number of migrasomes in each embryo was quantified. Zebrafish embryos treated with SAR407899 showed significantly reduced migrasomes number compared to zebrafish embryos treated with DMSO (Fig. 1f, g). It was reported that migrasomes formation in zebrafish embryos ensure the Kupffer’s vesicle (KV) formation which is essential in establishing the left–right body axis^3^. We found that treatment with SAR407899 indeed significantly impaired KV formation with smaller area and fewer cilia (Fig. 1h–j), further proving the effectiveness of the compound. Thus SAR407899 inhibited migrasome formation and further influence the KV formation in zebrafish.

SAR407899 inhibited migrasome formation both in cell culture and in zebrafish embryo, prompting us to conduct further mechanism studies. SAR407899 is an inhibitor for both ROCK2 and ROCK1. Knocking down ROCK2 did not interfere with migrasome biogenesis (data not shown) while knocking down ROCK1 significantly reduced the number of migrasomes generated per cell (Fig. 1k–m). It has been reported that ROCK1 knockdown impairs migration^7^. To study whether decreased migrasome formation was due to impaired migration, we further quantified the number of migrasomes per 100 μm of retraction fiber. In ROCK1 knockdown cells, the number of migrasomes/100 μm was significantly reduced compared to WT cells (Fig. 1n). Thus, reduced migrasome formation is not only due to migration defects. It has been reported that ROCK1 functions in cell adhesion to fibronectin^7,8^. Increasing the concentration of fibronectin increased the number of migrasomes formed in WT cells (Supplementary Fig. S1a), which indicates that migrasome formation is dependent on cell adhesion to fibronectin. ROCK1 knockdown cells did not sufficiently respond to increased fibronectin concentration compared to WT cells, suggesting that cell adhesion to fibronectin was impaired (Fig. 1o). We also used traction force microscopy to reconstruct and quantify the traction force created by the cells. Compared to WT cells, ROCK1 knockdown cells generated significantly less traction force (Fig. 1p, q). This suggests that migrasome formation is regulated by ROCK1 through its role in adhesion to fibronectin to generate a traction force.

Our study provides a pipeline for identification and verification of compounds and their protein targets in order to study the mechanism of migrasome formation. In the screening, 463 out of 507 compounds which decreased migrasome numbers in cultured cells decreased retraction fiber formation, indicating the effect of compounds in cell migration. This further confirmed that migrasome generation depends on cell migration. From the screening, we identified ROCK1 inhibitor SAR407899 which interfered with migrasome biogenesis without significantly reduced retraction fiber formation. ROCK1 regulated cell adhesion to fibronectin, which is an important factor regulating migrasome formation. In addition, we also identified the ROCK1 inhibitor SAR407899 which inhibited migrasome biogenesis in vivo in a zebrafish model system. Currently, more studies on the physiological and pathophysiological functions of migrasomes are being conducted in animal models. Thus, the identified compounds will provide tools to experimentally manipulate the biogenesis of migrasomes in physiological settings.

## Supplementary information

supplementary materiaal

## Supplementary information

**Supplementary Information** accompanies the paper at (10.1038/s41421-020-0179-6).
