# Supplementary material for: Chemical screening identifies ROCK1 as a regulator of migrasome formation
Source: Cell Discov. 2020 Aug 4;6:51. doi: 10.1038/s41421-020-0179-6 (PMC7400516; doi:10.1038/s41421-020-0179-6)
Supplement: Supplementary file 1 — supplementary materiaal [file 41421_2020_179_MOESM1_ESM.pdf]

Supplementary Fig. S1

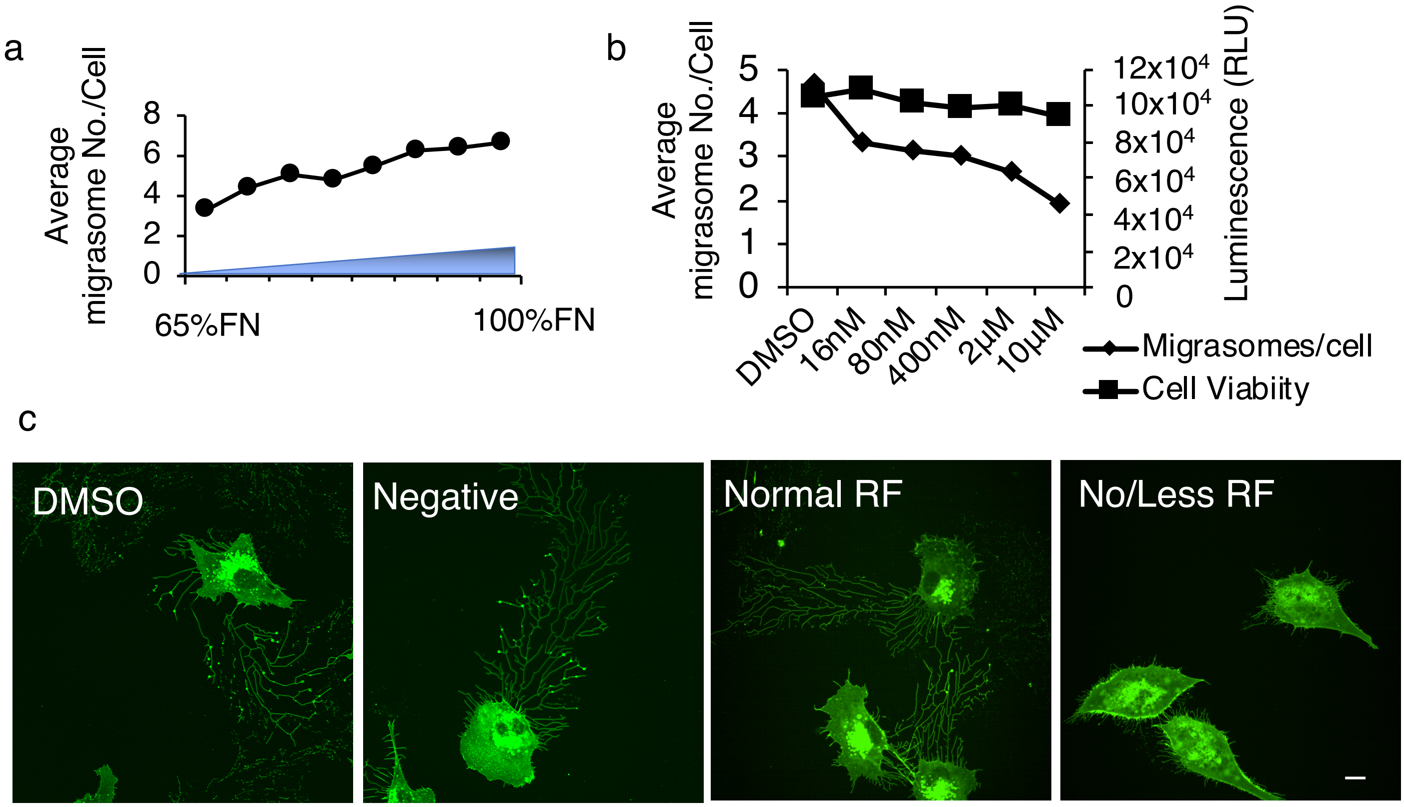

Supplementary Fig. S1 High-throughput phenotypic screen to identify compounds which inhibit migrasome formation

a. NRK cells expressing TSPAN-GFP were plated on glass-bottom dishes coated with an increasing concentration of fibronectin. Average migrasomes number per cell was quantified. 100%FN indicates that the culture plate was coated with 10 μg/ml fibronectin.

b. NRK cells expressing TSPAN-GFP were treated with different concentrations of GLPG0187. The average migrasome number per cell was quantified. Cells were also assayed for viability by measuring ATP content.

c. TSPAN4-GFP-expressing NRK cells were treated with chemical compounds from the screen. Representative images of different phenotype categories were selected. Scale bar, 10 μm.

Supplementary Fig. S2

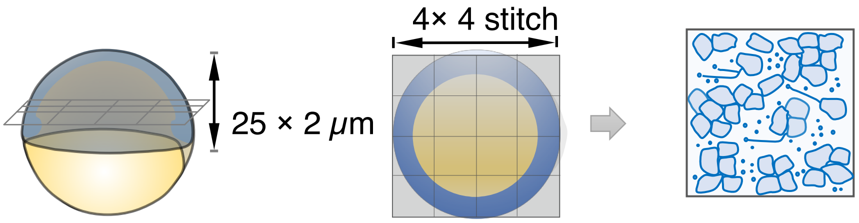

Supplementary Fig. S2 Characterization of SAR407899 on migrasome biogenesis in zebrafish embryos

Diagram showing zebrafish embryos for the quantification of migrasome number. Migrasomes labeled with PH–GFP were visualized in DMSO or SAR407899 treated embryos. The z-stack images of the embryos were acquired by spinning disk microscopy. 25 slices were acquired at 2-µm intervals from the animal pole. 16 images were combined for each slice.

Materials and methods

Cell lines and antibodies

NRK cells stably expressing TSPAN4-GFP were cultured in DMEM with 10% FBS (5% CO_2). For migrasome production, the culture dishes were coated with fibronectin (10 μg/ml) (PHE0023, Gibco) for 3 hours at 37 °C before the cells were seeded. Cells were seeded to 40% confluency in culture dishes for migrasome production. The antibodies used in this study are as follows: Anti-ROCK1 antibody (abcam, ab45171), Anti-αPKC (Santa Cruz, sc-216), anti-acetylated tubulin (Sigma, T6793).

Compound screening

A library of 2240 compounds with known protein targets was obtained from Shanghai Haoyuan. CellCarrier-96 well Microplates were coated with fibronectin (10 µg/ml) in PBS in a 37 °C incubator for 3 hours and the supernatant was removed by aspiration. NRK cells expressing Tspan4-GFP were then seeded into the wells of CellCarrier-96 well Microplates and cultured for 12 hours. For drug treatment in 96 well Microplate, 100 nl drug in DMSO (2240 drugs, 10 mM) was added into a new 96 well Microplate using Echo520 and then 100 μl cell culture medium was added into each well and thoroughly mixed with the drugs to make the final concentration of the drugs to 10µM. The mixture in each well was added to the corresponding wells of the plate seeded with cells to replace the original culture medium. The cells were incubated for 12 hours and then images of the plate were taken with a PerkinElmer Opera Phenix High Content Screening System. DMSO-only wells were used as a negative control.

Cell viability assay

The cell viability was determined using CellTiter-Glo®. Briefly, 100 µl CellTiter-Glo® Reagent was added to the 96-well plates. Mixing was carried out for 2 minutes on an orbital shaker and then the plates were incubated at room temperature for 10 minutes. Luminescence was then recorded using a plate reader.

Drug treatment of zebrafish embryos

WT zebrafish used in this study was from the Tuebingen (Tu) strain. PH domain-mCherry/GFP mRNA were injected at 4-cell stage to label migrasome. 10 nl of each drug at a dosage of 10 mM was injected into the space between chorion and embryo of 30% embryos. Then the embryos were incubated at 28.5 °C until the shield stage. For imaging, the embryos were dechorionated with proteinase and mounted with 1% low-melting-point agarose. Z-stack images of embryos were acquired by spinning disk microscopy at 2 μm intervals.

Whole-mount immunofluorescence of zebrafish embryos

Embryos at sphere stage were soaked in 200μM SAR407899 or equivalent DMSO till the 6-somite stage. The staining of zebrafish KVs were performed as previously described[3]. Briefly, embryos at the six-somite stage (12 h.p.f.) were fixed in 4% paraformaldehyde for 2 d at 4 °C. Then the fixed embryos were dehydrated in methanol and kept in methanol at –20 °C overnight. The dehydrated embryos were rehydrated in 0.5% Triton-X100 in PBS (PBST). The rehydrated embryos were blocked with goat serum for 1 h at room temperature and incubated with primary antibody anti-αPKC (1:50; sc-216, Santa Cruz) at 4 °C overnight on a slow shaker. The embryos were washed with PBST for 1 h each time for 3 times and incubated with secondary antibody anti-acetylated tubulin (1:50; T6793, Sigma) overnight at 4 °C on a slow shaker. The embryos were washed in PBST as before and finally mounted in 1% low-melting-point agarose for imaging.

Live-cell imaging

Cells were cultured in 35 mm glass-bottom dishes coated with fibronectin. Images were acquired using Olympus FV-1000 confocal microscopes.

siRNA sequences

The following siRNA sequences were used:
rat *Rock1* 5’-CCAAGUCACAAGCAGACAATT-3’;
NC siRNA 5’-UUCUCCGAACGUGUCAC GUTT-3’.

PAAm substrate preparation

Glass-bottom dishes (ϕ35 mm dish with ϕ20 mm glass bottom, In-Vitro-Scientific) were incubated with 10% 3-aminopropyltrimethyoxysilane (Sigma Aldrich) for 10 min and 0.5% glutaraldehyde solution (Beijing Chemical Works) for 30 min, rinsed by diH_2O and air-dried. A mixture of 10% acrylamide, 0.1% bis-acrylamide and 0.02% 200-nm red fluorescent microspheres (Thermo Fischer) were prepared as the stock solution. For polymerization, 1 µl of 10% ammonium persulfate (Sigma Aldrich) and 0.1 µl of TEMED (Sigma Aldrich) were added into 100 µl stock solution. After thorough mixing, 8 µl of the solution was pipetted onto an activated dish and a ϕ18 mm coverslip was carefully placed on it. The plates were left for 30 min for completion of polymerization. The Young's modulus of the substrate was 10.61 kPa. To facilitate cell adhesion, the substrates were coated with fibronectin at 10 µg/ml using sulfo-SANPAH (Thermo Fischer) crosslinker according to a previously published protocol by Tse and Engler.

Traction force microscopy

TSPAN4-GFP-expressing NRK cells treated with NC or *Rock1* siRNAs were plated on PAAm substrates for 12 h. Images of fluorescent beads and adherent cells were taken before and 5 min after applying 1 ml of 1 M NaOH to detach cells.

Open source MATLAB GUI software was used to process the data. Correlation-based particle tracking velocimetry (PTV) was utilized to quantify the displacement field of the microspheres on the substrates corresponding to the cell-induced substrate deformation. The displacement field was interpolated onto a regularized grid and the stress field was calculated by regularized-Fourier transform traction cytometry (reg-FTTC). The regularization parameter was set as 10^{-5}.

Statistics

Statistical analysis was performed in GraphPad Prism. All data were obtained from at least 3 independent experiments. Error bars in the figures represent the mean ± s.e.m. Comparisons were performed by two-tailed, unpaired Student's t-tests. $P < 0.05$ was considered statistically significant.
